# Highlights of neuroanatomical discoveries of the mammalian gonadotropin‐releasing hormone system

**DOI:** 10.1111/jne.13115

**Published:** 2022-05-03

**Authors:** Rebecca E. Campbell, Lique M. Coolen, Gloria E. Hoffman, Erik Hrabovszky

**Affiliations:** ^1^ Centre for Neuroendocrinology and Department of Physiology, School of Biomedical Sciences University of Otago Dunedin New Zealand; ^2^ Department of Biological Sciences Kent State University Kent Ohio USA; ^3^ Department of Biology Morgan State University Baltimore Maryland USA; ^4^ Laboratory of Reproductive Neurobiology Institute of Experimental Medicine Budapest Hungary

**Keywords:** human, luteinizing hormone‐releasing hormone, mouse, reproduction, sheep

## Abstract

The anatomy and morphology of gonadotropin‐releasing hormone (GnRH) neurons makes them both a joy and a challenge to investigate. They are a highly unique population of neurons given their developmental migration into the brain from the olfactory placode, their relatively small number, their largely scattered distribution within the rostral forebrain, and, in some species, their highly varied individual anatomical characteristics. These unique features have posed technological hurdles to overcome and promoted fertile ground for the establishment and use of creative approaches. Historical and more contemporary discoveries defining GnRH neuron anatomy remain critical in shaping and challenging our views of GnRH neuron function in the regulation of reproductive function. We begin this review with a historical overview of anatomical discoveries and developing methodologies that have shaped our understanding of the reproductive axis. We then highlight significant discoveries across specific groups of mammalian species to address some of the important comparative aspects of GnRH neuroanatomy. Lastly, we touch on unresolved questions and opportunities for future neuroanatomical research on this fascinating and important population of neurons.

## 1 A BRIEF HISTORICAL OVERVIEW

A major force in the understanding of systems neuroscience and the system regulating reproduction arose from the concept that “function follows form.” Studies of neuron morphology reveal changes during development, maturation, senescence, experience and plasticity following injury. Information about these important components leads to understanding of normal function and how dynamic changes alter the way the system works. For neuroendocrinology, anatomy has had a seminal role. First and foremost, discovery of neurosecretion and its unique role in endocrine processing changed the perspective of the roles that neurons play in homeostasis and brought to light how neurons can work as secretory units directed into the peripheral circulation. The acceptance of the concept of neurosecretion arose from the results of anatomical studies that began in the late 1930s and extended through the 1960s. Those studies established that neurosecretory material originated from neurons that extended their axons from the brain to the posterior lobe of the pituitary. The neurohormones released from the axon terminals near fenestrated blood vessels were transported by the blood circulation to distant sites. As the cited reviews indicate,^1^, ^2^ these seminal studies initiated the field of neurosecretion and provided key insights into the unique roles of neuroendocrine neurons in the control of both posterior and anterior pituitary functions. However, the study of the reproductive neuroendocrine system would still be a black box had it not been for the discovery of GnRH. There is little doubt that the award of the Nobel Prize to Andrew Schally and Roger Guillemin in 1977 set the stage for unravelling the mysteries of reproductive physiology.

For the control of anterior pituitary‐related functions (regulation of growth hormone, thyroid hormone, gonadotropin, prolactin and corticosteroids secretion), anatomical studies led to the primary role of the median eminence and pituitary stalk as the sites of factor release. The unique blood vessels forming loops within the median eminence^3^ were found to collect into “private” vessels that led exclusively to the anterior pituitary to control adenohypophyseal functions. As evidence mounted suggesting that the brain made the factors regulating the anterior pituitary, anatomical studies describing the reproductive function upon transplantation of the anterior pituitary into the medial basal hypothalamus (MBH)^4^, ^5^ compared to the kidney capsule led to the initial evidence of where the neural control systems resided. They emphasized the need for the pituitary gland to have a close proximity to the hypothalamus and its portal blood system and that placement of median eminence extracts into the pituitary enabled ovulation.^4^, ^5^, ^6^, ^7^, ^8^ While these early studies indicated that the anterior pituitary needed close proximity to the median eminence, data did not reveal where the brain cells providing the stimulating factors for gonadotropins resided.

Use of reproductive function assays (ovulation or eventually measurement of LH or FSH) after brain stimulation^9^ or knife cuts placed stereotaxically into the brain^10^ began to suggest that reproductive control in a number of species required an intact preoptic area (POA) connection to MBH. While it is beyond the scope of this article to review each of those studies in all the species that have been used with these approaches, one must be cognizant of the fact that interpretation of studies using knife cuts, lesions or electrical stimulation were impaired or easily misinterpreted when the locations of the system and its pathways were not yet defined. An understanding of where the GnRH neurons were located and where their axons projected were required for a deeper understanding of how the neurons controlled LH and FSH secretion. In the 1970s, as antibodies against GnRH were produced and techniques for visualizing GnRH became available, scientists began to search for the elusive GnRH neurons. Few of those early studies were successful due to suboptimal tissue processing conditions and antibody qualities. Investigators tried to apply the same preparations for hypothalamic tissue that were used for routine histology both in terms of selection of fixatives and preparation of tissue blocks in paraffin for cutting. GnRH was easily extracted during tissue dehydration in EtOH and embedment in paraffin which resulted in a striking loss of the GnRH antigen in paraffin‐embedded material. If embedding in paraffin was avoided by processing free‐floating sections, staining greatly improved.^11^ Fixation known to be optimal for routine histology (Bouin's) included formaldehyde, picric acid and acetic acid and had a pH close to 1.0. It turned out that staining of tissue fixed in a neutral formaldehyde ‐ picric acid fixative (Zamboni's) preserved GnRH better and provided clear locations of the cells and their axons compared to staining of tissue fixed with the more acidic fixative. Moreover, initial recommendations for fixation of peptides suggested short fixation times and cold solutions. Missed was the fact that formaldehyde‐based fixatives are not able to complex proteins quickly. If the fixative was cool, the process became even slower. It was later learned that fixatives that penetrated better and reacted more readily than buffered formaldehyde/formalin fixatives markedly improved the detection.^12^ Once GnRH antisera with high titres were produced or purified after production, antigen detection improved further. Scientists could then distinguish immunoreaction products and details of cell morphology. It is essential, however, that one considers the method selected for detection. Methods that generate few molecules of product not only require more antibody but may disable detection of structures with lower expression. Comparisons of methods^13^ reveal the striking differences in antigen detection, especially using immunofluorescence approaches.

Early studies often assumed that the cells of the GnRH system would be found in the MBH. Some may not have probed carefully other brain areas or based their conclusions on assays of extracts that showed high activity in the MBH, in virtually all species. Some attempts to stain those cells had used an antibody later found contaminated by proopiomelanocortin antibodies. Arcuate nucleus (ARC) stained cells ended up being opiate cells since the antibodies had been repurposed after first immunizing the rabbits with proopiomelanocortin peptides and not GnRH.^14^ As localization improved it was realized that the high GnRH concentrations in the ARC were due to the axons converging into the median eminence rather than cells (if present in the area, the cells were often positioned lateral to the ARC). Once descriptions of cell locations accumulated, locations widely scattered in the forebrain seemed to contradict the notion that neurons developed from the ventricular surfaces and migrated to their final destination in a programmed fashion like most other CNS neurons. GnRH neurons were different. Diverse species were found to have GnRH neurons in different sites, and initially this made no sense. It was then the seminal studies of Schwanzel‐Fukuda and Pfaff^15^ and Wray et al.^15^ both in 1989, that lit the light bulb indicating those neurons developed from the olfactory placode unlike most neurons of the central nervous system. That discovery also paved our way for understanding how genetic disorders of olfactory placode cell migration linked anosmia to reproductive dysfunction (Kallmann's Disease). Once the manner in which the olfactory system generated GnRH neurons was uncovered^15^ we could better recognize that GnRH cells all fell into a part of what we now know as the rostral migratory stream. Table 1 shows site variation in a variety of species.^16^ Note there are vast differences in the location of cells, ranging from small ganglia at the interface of the olfactory nerve whose cells extended into the rostral septum in the opossum,^17^ to the midbrain in primates and dogs. Cells were positioned in the lateral anterior tuberal hypothalamus (along the lateral borders of the ARC) in species where the forebrain is vertically shifted (primates, sheep, ferrets and guinea pigs), whereas a dominance of medial POA and septal cells were found in rodents, cats, hamsters, voles, and pigs. Nonetheless, there are a small number of GnRH neurons in the latter group that migrate into the arcuate nucleus and sometimes the pituitary stalk along with the axons from more rostrally positioned neurons. While not yet known why the cells move further along the migratory stream in some but not all species, the association of the neuroendocrine members of the GnRH system do seem to follow the known roads from the nose into the brain.^16^ A feature of the GnRH cells that travel into more caudal brain areas is that they often reside between rather than within the resident nuclei. This phenomenon might be explained by the fact that in those animals, GnRH neurons arrive in those areas at the time when the nuclei have already formed. For example, in the rat, migration of GnRH neurons is completed by embryonic day 16.5,^18^ whereas at that time, neurons of the medial POA have already left the mitotic cycle and occupy their adult locations.^19^

**TABLE 1:** Diversity of distribution of GnRH neurons in mammals. Note these data are derived from use of antibodies that appeared not to stain truncated or modified forms of GnRH. Modified from Hoffman and Berghorn^16^

	Septum	Preoptic area	Anterior hypothalamus	Tuberal hypothalamus	Premammalian
Rodents					
Mouse	++	++++	++	+/−	−
Hamster	+++	++++	+	−	−
Rat	++	++++	++	+/−	−
Guinea pig	+	+++	++	+	−
Lagomorphs					
Rabbit	++	+++	+	+	+
Chiroptera					
Bat	−/+	−/+	+	++++	−
Carnivores					
Ferret	−/+	+	+	++++^a^	−
Dog	+	++	++	++	+
Cat	+	+++	++	−	−
Ungulates					
Sheep	++	++++	+	+	−
Pig	++	++++	+	−	−
Marsupials					
Opposum	+/−	−	−	−	−
Primates					
Rhesus monkey	++	++++	+++	+++^a^	+
Baboon^a^	++	++++	+++	+++^a^	+

^a^ Lateral to the Arcuate nucleus.

The fact that the axons of GnRH neurons reached the pituitary portal blood supply provided an opportunity for anatomy to reveal which of the GnRH cells have the ability to influence the pituitary. In the late 1980s and 1990, application of retrogradely transported molecules administered into the median eminence, and taken up by the axons of the portal blood supply or injected into the peripheral circulation, gave insight into which GnRH cells were potentially able to release GnRH into the peripheral circulation (horseradish peroxidase,^20^ wheat germ agglutinin,^21^ and fluorogold^22^, ^23^). In all those studies, only a portion of the GnRH neurons possessed the tracer. The fact that some GnRH cells lacked connection to the median eminence is noteworthy in that it stresses the fact that subsets of GnRH neurons have a neuroendocrine role not shared by all GnRH cells in the area. Comparisons across species speak to a similarity of subsets of neuroendocrine GnRH cells in mice, guinea pigs, sheep, and primates.

Scientists were then faced with the question of whether examining the GnRH neuron architecture could provide insight into the transitions across reproductive status. Do the cells visibly change during the transition to puberty or senescence? Overall, the data are inconsistent and with high variation in the quality of staining. When changes are observed, such as development of spines in rats during adolescence as an indicator of the pubertal period, some of those changes do not accurately reflect the functional status of the GnRH cells^24^, ^25^ but may instead reflect the capacity to cycle rather than the actual presence of altered cyclic hormone function. In contrast, studies in mice elegantly revealed that at the time of activation of GnRH neurons spines were increased by 60% over quiescent times.^26^ Studies of this type address the plasticity of spines on neurons and emphasize the importance of knowing the details of the animal's physiological status when probing for morphological changes.

**FIGURE 1:**
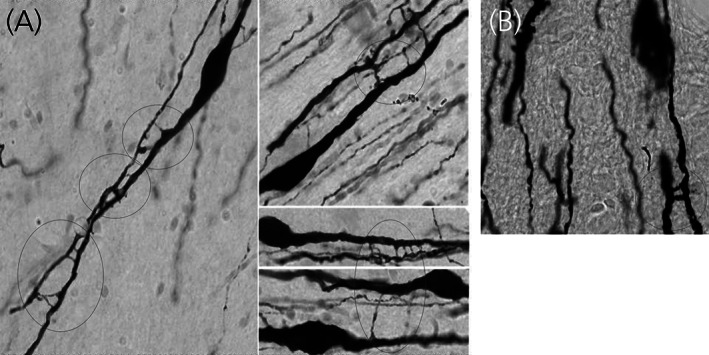
Dendrodendritic interactions of GnRH neurons. Intercellular bridges are seen between GnRH dendrites. (A) Examples of dendritic processes from a rhesus monkey whose bridges between immunoreactive GnRH neurons appear as fine ladders. (B) Example of two intercellular bridges across GnRH neurons in a rat brain (red circle). Figure reproduced from Hoffman^33^

Where neuroanatomy has been valuable in reflecting functional changes in the GnRH system arose from the observations that many neurons, when stimulated, express immediate early gene proteins. FOS is one of these proteins and has the advantage that it is expressed within 30–45 min after the cells are stimulated. Fortunately, the FOS protein is also highly conserved across species. The induction of FOS is the consequence of sharp changes in second messenger signals arising from synaptic activity. Baseline cell firing alone often cannot reach the threshold for FOS induction. FOS has enabled the identification of those GnRH neurons that give rise to the preovulatory LH surge in female rats, mice, sheep, and ferrets. In rats, taking into account the time it takes for FOS translation (30–60 min), the number of cells displaying FOS is tightly coupled to the rise in LH that triggers ovulation.^27^ In these species FOS is not triggered by hormonal signalling in males. This reflects the absence of the oestrogen‐sensitive cells driving GnRH secretion.^28^ Surprisingly, a study attempting to show that FOS identified activated GnRH neurons in primates during the preovulatory LH surge did not show evidence of activation of the GnRH system.^29^ However, the primate LH surge persists for days due to pituitary drive of LH. Also, since FOS will only remain in stimulated cells for 2–3 h after onset of stimulation the fact that the time of examination was not linked to the onset of LH secretion could explain why the cells did not show signs of recent stimulation. Indeed, examination of GnRH release from the median eminence of cycling female rhesus monkeys showed a marked surge in GnRH at the time of the preovulatory LH surge.^30^ What this reinforces is that unless accompanied by careful monitoring of LH secretion at the onset of LH surges, FOS can appear unreliable as an activity marker. Another consideration is the fact that FOS induction does not persist, but rather initial induction of second messenger signals turns on the *c‐fos* gene. FOS then appears to turn off its own mRNA production and protein turnover stops the signal within a couple of hours even when stimulation persists for days. Only after the system normalizes can FOS be induced again. Repeated surges of LH if separated by 24 h permit the re‐expression of FOS in the GnRH neurons.^31^ An important problem unresolved for a long time was how GnRH neuronal function enables GnRH pulsatile release at times other than the preovulatory surge. Both males and females in multiple species show pulses of GnRH and LH that are spaced between 30 and 60 min apart. It has been suggested that pulses might simply represent the random firing of two GnRH neurons, but the timing of pulsatile release seems too regular for events to be random. Over the years it has also been hypothesized that GnRH–GnRH synapses (axodendritic, dendrodendritic) could link cell activity or that gap junctional dendrodendritic connections suggested by Witkin et al.^32^ from intercellular bridges could provide synchronous activity between GnRH neurons. An additional explanation for the function of the dendritic interactions could be ephaptic transmission but the diffuse organization of the cells and challenge of knowing which cells should be assessed have made it difficult to determine the nature of connections that underlie the pulses. The interaction of cellular processes in GnRH neurons is seen in both primates and rat and could be the result of dendron or dendritic interactions in mice. Examples of dendritic associations in a primate and a rat is presented in Figure 1.^33^ The advent of genetic animal models expressing reporter proteins under the control of the GnRH promoter has since richly contributed to our understanding of the connectedness of GnRH neurons. As discussed in detail below, the absence of electrical coupling between GnRH neurons was initially reported in mice expressing GFP in GnRH neurons,^34^ and later confirmed in other reports.^35^ Evidence accumulated in recent years now indicates that kisspeptin (KP) release from MBH KP neurons which cosynthesize KP, neurokinin B (NKB) and dynorphin (Dyn; aka “KNDy neurons”), plays a pivotal role in shaping the patterned secretion of GnRH into the hypophysial portal circulation.^36^, ^37^

Another challenge in the studies of the GnRH system is with little doubt that while the male control of gonadotropin section is vastly different from that of the female, scientists have found little evidence of GnRH neuron sexual dimorphisms. In that domain, a major clue arose from the fact that the alpha oestrogen receptor isoform (ER‐α) was essential for cyclic release of GnRH and gonadotrophins, but GnRH neurons had no ER‐α.^38^ Further investigation of oestrogen's site of action led to the KP neurons which not only possess ER‐α but also display striking sexually dimorphic distributions.^39^

## 2 SPECIES SPECIFIC GNRH NEUROANATOMY: RODENTS

**FIGURE 2:**
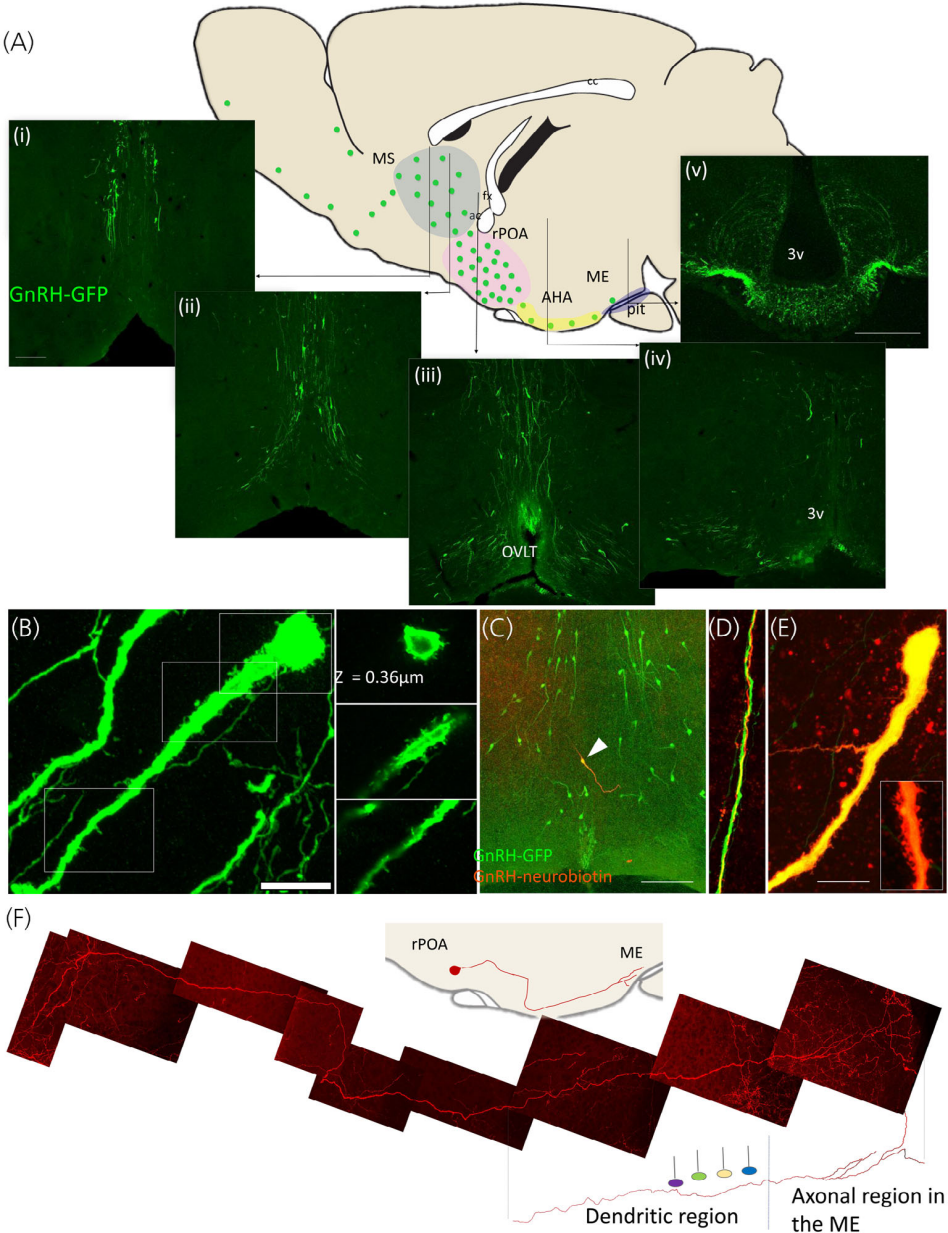
Transgenic approaches to reveal GnRH neuron morphology in the rodent brain. (A) A sagittal brain schematic depicts the scattered distribution of GnRH neurons in a mouse brain (green dots). Coloured regions correspond to commonly referenced anatomical zones, that is, the medial septal region (MS, blue), the rostral preoptic area (rPOA, pink) and the anterior hypothalamic area (AHA, yellow). Vertical lines indicate the location of representative confocal images in the coronal plane of a GnRH‐green fluorescent protein transgenic mouse (i–v). (B) GnRH neuron spines can be visualised in confocal images of the GnRH‐GFP mouse brain. Fixed labelled brain sections and following neurobiotin cell‐filling in ex vivo brain slices from GnRH‐GFP mice. Bar = 10 μm. (C) Neurobiotin filling of GnRH neurons in GnRH‐GFP muse brain slices reveals long dendritic projections, found to bundle with other GnRH dendrites (D) and exhibit proximal and distal spiny protrusion (E). (F) An individual GnRH neuron originating in the rPOA and terminating in the median eminence can be visualised through sparse viral mediated transfection of membrane linked fluorophores in GnRH‐Cre transgenic mice coupled with optical clearing techniques. Together with functional evidence, the long dendritic processes of GnRH neurons that transition into axonal processes supports the presence of a blended dendron. Bar = 100 μm in (A) and (C) and 10 μm in (B) and (E); fx: fornix, ac: anterior commissure, cc: corpus callosum. OVLT: organum vasculosum of the lamina terminalis, 3v: third ventricle, ME: median eminence, pit: anterior pituitary gland. Parts of figure adapted from Campbell^51^

The first visualisation of GnRH neurons with immunohistochemistry was reported in the guinea pig^40^ (reviewed in^41^) and described as a diffuse distribution of bipolar neurons and their fibre processes through the basal forebrain. Since that time, the distribution and morphology of GnRH neurons has been described and studied most frequently in the rat^42^, ^43^ and mouse,^44^ but also in several other rodent and small mammal species, for example the hamster,^45^, ^46^, ^47^ bat,^48^ and rabbit,^49^ and in larger mammal species (see further below). In general, rodent GnRH neurons are found dispersed throughout the septal, preoptic and anterior hypothalamic areas.^50^ They are evident along a rostral to caudal continuum, from the olfactory bulbs to the medial basal hypothalamus, following their developmental migratory path into the brain.^15^, ^18^ In the rat and mouse, the diffuse somata of GnRH neurons are frequently described by three anatomical subpopulations, including a rostral most population that is found to run in dorsoventral bilateral streams from the medial septum to the rostral POA, a central population in an inverted Y distribution around the organum vasculosum of the lamina terminalis (OVLT), and a smaller lateral population that sits ventrally on both sides of the third ventricle in the anterior hypothalamus (Figure 2A). While it is not clear whether GnRH neurons residing in different locations possess specialised functions, a large proportion of those neurons surrounding the OVLT colabel with the immediate early gene marker FOS at the time of the LH surge in rodents,^52^ suggesting that this subpopulation of neurons is particularly important in surge generation. GnRH neuron axon processes in rodents can be visualised along the rostral to caudal continuum of the GnRH neuron soma, extending into the OVLT and densely infiltrating the external zone of the median eminence. GnRH projections have also been described in a number of hypothalamic and extra hypothalamic regions outside of this zone, including the olfactory system, amygdala, limbic cortex and midbrain^45^, ^53^, ^54^ supporting the notion for extra‐hypophysiotropic roles for GnRH neurons as a central nervous system neurotransmitter.

Studies of GnRH neuron morphology in rodents, through both classical and transgenic approaches, have advanced and challenged our understanding of GnRH neuron function. Collectively, these anatomical discoveries have formed a foundation for understanding how GnRH neurons are regulated by upstream networks, behave across different physiological states, and operate in a synchronized fashion for pulsatile release.

### 2.1 Distinct regulation zones

As the final downstream effector neurons driving the central regulation of fertility, a large focus has been on understanding how GnRH neurons are regulated by higher order inputs. Early ultrastructural studies described GnRH neuron somata and proximal dendrites in several species as largely ensheathed by glial membrane and very sparsely innervated relative to surrounding neurons.^42^, ^46^, ^55^, ^56^ These early studies promoted the idea that the integration of synaptic input required for successful pituitary‐gonadal axis control probably occurs upstream from the GnRH neurons themselves. This idea has been both challenged and reframed with the development of new ways to visualise GnRH neuron morphology and the discovery of the KP system.

Filling murine GnRH neurons in brain slices with low molecular weight dyes^57^, ^58^, ^59^ or expressing membrane bound fluorescent markers in small numbers of GnRH neurons in vivo^60^ has expanded our view of GnRH neurons beyond regions limited by GnRH peptide detection (Figures 2C–E). GnRH neurons have been found to extend exceptionally long processes that possess dendritic morphology, suggesting large anatomical zones important in receiving and processing afferent information. Most GnRH neurons extend two of these long dendrites, and in many cases, both project ventrally and caudally toward the median eminence, with dorsally oriented dendrites frequently taking a hairpin turn to follow other projection toward the median eminence.^61^, ^62^ Labelling for ankyrin G has identified the axon initiation zone in one of these dendrites, typically 50–150 μm away from the soma.^61^ Histological evidence for synaptic inputs places the majority of inputs within this dendritic zone between the soma and the axon initiation zone,^58^, ^61^, ^63^ and quantification of apposed vesicular transporters suggests that glutamatergic inputs are twice as likely as GABAergic inputs within this zone.^63^ In addition, the number of putative synapses to the GnRH neuron proximal dendrite drops precipitously beyond about 50 μm.^58^, ^63^ These data suggest that although the GnRH neuron dendrite can extend for millimetres through the mouse brain, there is still a concentrated area in the proximal dendrite where the majority of synaptic input is received, at least at this “end” of the GnRH neuron.

Cell‐filling and reporter expression in GnRH neurons also discovered that as dendrites approach the ME, they branch into multiple processes with axonal morphology that extend to and terminate on blood vessels^60^, ^62^ (Figure 2F). This was not entirely new, as early morphological studies also described dendritic projections that appeared to transition into axonal projections.^42^ More recently, the blended dendritic and axonal features of this process, have been confirmed with electrophysiology and electron microscopy,^62^ and led to the term “dendron” (reviewed in Herbison^64^). Morphological evidence suggests that this distal region of the GnRH neuron is much more densely innervated than the proximal dendrite,^60^, ^65^ and that synaptic inputs are restricted to the dendritic portion of the dendron and absent from axons.^60^ Expansion microscopy suggests that dendrons are also responsive to short‐diffusion volume transmission, as local KP‐expressing axons do not appear to make synaptic contacts with the dendron.^66^ Evidence for a dendron‐like structure has also been described in the rat,^67^ although ultrastructural studies will be required to confirm this morphology.

The identification of these two distinct input zones, at opposite poles of the GnRH neuron, has evolved alongside the discovery of two distinct anatomical and functional populations of KP neurons in the mouse. Selective viral‐mediated tracing has identified that the most rostral population of KP neurons, associated with surge generation, project most heavily to the proximal zone of GnRH neurons, while the KNDy neurons of the ARC, the putative pulse generators,^68^ project exclusively to the distal zone.^69^ This morphology, supported by functional evidence,^65^ suggests that the proximal zone is critical for surge generation and the distal dendron is a separately regulated domain critical for pulsatile GnRH secretion.

To date, evidence for a GnRH dendron‐like process has only been described in the mouse,^62^ and the rat^67^ and it remains unclear whether the same feature is exists in other mammals. Of interest, the GnRH neurons of the small brown bat, which have a distribution that is more similar to the primate pattern than the rodent pattern, is described to have one thin and one thicker process leaving the soma, both of which give rise to fine varicose fibres.^48^ Presumably the thicker process is dendritic in nature and the varicose fibres are axonal, describing a similar morphology to that seen in the mouse. Another clue that this feature may be more universal comes from the ewe, where median eminence explants are found to respond to KP,^70^ suggesting that distal GnRH neuron elements are responsive to neurotransmission.

### 2.2 Function‐related morphological plasticity

Visualisation of rodent GnRH neurons has repeatedly demonstrated morphological plasticity associated with changes in circuitry, function and physiological state. Morphological plasticity of the GnRH neuron soma and proximal dendrite was suggested by early immunodetection studies reporting somatic profiles of GnRH neurons as “smooth” or “thorny.” These shape‐types were found to vary with age and hormonal status^71^, ^72^, ^73^ and with the number of ultrastructurally identified synaptic inputs,^42^ suggesting that these ‘spines’ were presumptive sites of excitatory input, as in other neuronal populations.^74^, ^75^ Later studies, using transgenic mice and rats expressing green fluorescent protein (GFP) in GnRH neurons,^34^, ^76^, ^77^, ^78^ afforded an enhanced view of spine morphology (Figure 2B) and found the density of these putative spines to increase in several physiological states, including over pubertal development,^79^ in FOS activated neurons at the time of the preovulatory LH surge,^26^ and in association with hyperactivity in prenatally androgenised mice.^80^ Although the presence of these spines correlates with physiological states in which greater synaptic inputs may be anticipated, and in some case has been shown,^81^ there is currently limited evidence to support that the spines identified on GnRH neuron soma or dendrites are indeed sites of synaptic input or reflect morphological rearrangements in synaptic inputs, in particular over the female oestrous cycle.^63^, ^82^ In contrast, seasonal breeders, including the ewe and hamster, have been found to exhibit dramatic plasticity synaptic input density to GnRH neurons.^83^ It remains unknown whether GnRH neurons in these species possess spines similar to the rat and mouse and whether they exhibit morphological plasticity. To date, all evidence for morphological plasticity comes from the proximal input zone of the GnRH and it remains to be determined whether morphological plasticity occurs in the distal region of the dendron.

The nerve terminal zone of rat GnRH neuron axons has also been well described to undergo morphological plasticity to facilitate hypophysiotropic peptide release into the perivascular space.^84^ Ultrastructural studies show GnRH nerve terminals in the external zone of the median exhibit neuro‐glial remodelling over the estrous cycle, dependent upon the actions of semaphorin 7A expressed in local tanycytes.^85^ Similar structural rearrangements have been shown in the ewe between the breeding and anoestrus seasons^86^ and with altered metabolic status.^87^

### 2.3 Dendritic bundling: A morphology to support synchronisation or simply a product of migration?

Ultrastructural evidence for closely associated GnRH neuron dendrites and shared synaptic input (a single axon synapsing with two GnRH neuron dendrites) was first described in rats in the mid 80s.^88^ More recently, colabelling of biocytin‐filled GnRH neurons and GFP in brain tissue from GnRH‐GFP transgenic mice also identified several examples of these ‘bundled’ GnRH neuron processes, where the dendritic processes of 2–4 distinct GnRH neurons were found intertwining and coming in close contact with one another^89^ (Figure 2D). At the ultrastructural level, these bundles are found frequently connected via zonula adherens and contacted by shared synaptic input from single afferents.^89^ While tempting to speculate that GnRH neurons are electrically coupled via these close associations to support synchronised activity, GnRH neurons do not express gap junctions.^35^ This suggests that the main function of these bundles may serve as sites for efficient innervation by upstream regulators, aimed at simultaneously targeting several GnRH neurons. To date, these elements have only been described for more proximal regions of the GnRH neuron dendrite and it remains to be determined whether similar features exist in more distal regions where pulse generation appears to be predominantly regulated.^65^ While postulated as a potential morphological substrate for synchronisation, an alternative hypothesis is that the bundling morphology of GnRH neurons is a byproduct of early migration and dendritic pathfinding. While dendritic bundling suggests a mechanism by which the largely scattered rodent GnRH neurons can be in physical contact with one another, it remains to be determined if or how this morphology is important for GnRH neuron function.

## 3 SPECIES SPECIFIC GNRH NEUROANATOMY: SHEEP

Early work in the sheep provided some of the seminal observations characterizing the morphology of GnRH neurons and their neuroendocrine functions. These include initial observations of the close, intimate anatomical relationship of GnRH neurons with surrounding glia, as well as evidence suggesting regional differences among GnRH subpopulations in their activation during pulsatile versus surge secretion of LH. In addition, because sheep are seasonal breeders, they have provided a model demonstrating seasonal plasticity of inputs onto GnRH neurons, work that has more recently been extended into plasticity of specific sets of afferents. Finally, it is worth noting that because of the unique ability in sheep to directly monitor GnRH in portal blood in awake animals,^90^ it has been possible to directly correlate patterns of GnRH neuronal activity using FOS and other markers with their neurosecretory output. In the following sections, we will briefly review some of these key observations, and how they formed a foundation and, in some cases, presaged more recent findings.

### 3.1 GnRH neuron morphology and projections in sheep

Early immunocytochemical work using free‐floating, unembedded sections, and including the use of detergent (Triton‐X100) in processing, revealed a complex morphology of GnRH neurons in the sheep brain, with significant numbers of multipolar neurons^91^ in contrast to the predominantly bipolar morphology of GnRH neurons in rodents (see above). While the majority of GnRH neurons in the sheep were seen in the POA, smaller but significant numbers were seen more caudally in the anterior hypothalamus and MBH.^91^, ^92^ Subsequent tract tracing studies in sheep,^93^ similar to such studies in rodents, showed that despite the regional variation in GnRH cell number, the percentage of GnRH cells in each area projecting to the median eminence remained consistent across regions, suggesting that GnRH neurons in all areas were capable of controlling the release of pituitary LH. Perhaps the most remarkable observation of these early studies in sheep was the degree to which GnRH cells, unlike adjacent non‐GnRH neurons, were found to be almost entirely ensheathed by the processes of adjacent astrocytes.^94^ Afferent axon terminals were seen to penetrate this glial sheath before synapsing upon preoptic GnRH somata and dendrites.^94^ This observation set the stage for subsequent studies of plasticity of inputs to ovine GnRH neurons, related to neuroendocrine control by season^86^ as well as alterations in the adult GnRH system due to prenatal androgen treatment.^95^ Interestingly, it also complemented work in rodents documenting the loose functional relationship between glial and GnRH cells in sexual maturation, including recent demonstration of mechanisms by which GnRH neurons “recruit” astrocytes in postnatal development to ensure appropriate wiring and firing of GnRH neurons.^96^

### 3.2 Seasonal plasticity of the GnRH system in sheep

Sheep and other seasonally breeding mammals display a reversible annual cycle of fertility in which GnRH neurons are the critical, final common output controlling reproduction. In sheep and most other mammals, the key factor responsible for seasonal reproduction is a profound change in the ability of estradiol to inhibit pulsatile secretion of GnRH.^97^ The early recognition that GnRH neurons in sheep and other species lacked nuclear ER‐alpha,^98^ the isoform required for oestradiol's negative feedback influence, implied that the influence of estradiol must be conveyed to GnRH via afferents. Given the striking degree of glial ensheathment of GnRH cells in the sheep, it was logical to compare the ultrastructure and synaptic inputs of GnRH neurons between the breeding and nonbreeding (anoestrous) animals as a possible structural basis for seasonality. The results of an incredibly labour‐intensive, electron microscopic analysis revealed that GnRH neurons in the breeding season received more than twice the density of synaptic inputs onto their dendrites and somas.^86^ These changes in synaptic density were complemented by a reciprocal change in the amount of glial ensheathment of GnRH cells, with cells in anestrous animals being more completely surrounded by glial processes. Importantly, both the changes in synaptic input and glial ensheathment were only in GnRH cells and not in adjacent nonidentified neurons in the POA. Later studies provided details of the neurochemical phenotype of the seasonally changing inputs,^99^ including work showing increased number of KP, and decreased GnIH, contacts into GnRH neurons during the breeding season.^100^ This demonstration of seasonal plasticity in the GnRH system also provided a conceptual foundation for other studies documenting changes in the number and phenotype of afferents onto adult GnRH neurons, including studies of changes associated with the preovulatory GnRH/LH surge,^101^ and long‐term changes seen as a result of prenatal androgen exposure in female sheep.^95^, ^102^ Finally, seasonal synaptic changes in the reproductive neuroendocrine system of sheep are not limited to GnRH neurons, but also seen in inputs onto upstream afferent neurons, notably KNDy cells of the ARC.^103^ Upstream changes in the number and density of synaptic inputs onto KNDy cells are also seen in prenatally androgenized ewe^95^, ^102^ and prenatal androgen mouse^104^ models of polycystic ovary syndrome (PCOS).

### 3.3 Functional subpopulations of GnRH neurons in sheep

As discussed above, when used in conjunction with monitoring of secretory LH patterns, FOS has been a useful marker for GnRH neuron activation in a number of species. This has been particularly true in the sheep, where discrete and repeated measurements of portal GnRH are possible over long periods of time in unanaesthetized animals.^90^ Indeed, the ability to precisely monitor the dynamics of GnRH pulses and surge has allowed for examination of the activation of GnRH and other upstream neurons (e.g., KNDy neurons) during both pulsatile^105^, ^106^ and surge secretion.^92^, ^101^, ^107^ Whereas about half of all GnRH neurons across the entire range of their distribution, including the POA and MBH, expressed FOS during the preovulatory LH surge,^107^ using two models that acutely induced an LH pulse, FOS expression was seen to be limited to GnRH cells located in MBH.^105^ Interestingly, in the latter experiment, the occurrence of endogenous (not induced) LH pulses was also accompanied by FOS activation in MBH but not POA GnRH neurons.^105^ More recent work in the sheep has reinforced the view that MBH GnRH neurons are functionally involved in pulse generation. First, unlike POA GnRH cells, they are heavily innervated by Dyn terminals^108^ presumably arising from KNDy neurons which constitute a core component of the GnRH pulse generator.^36^ In addition, recent work using internalization of kappa opioid receptor as a marker has shown that Dyn is released onto MBH, but not POA, GnRH neurons at the time of the termination of each GnRH pulse.^103^ Thus, at least in sheep, there is strong evidence for the most caudal subpopulation of GnRH neurons in the MBH being selectively involved in pulsatile secretion, perhaps in parallel to actions at the GnRH “dendron”, also located in the MBH, as a locus of pulse regulation in rodents.

## 4 SPECIES SPECIFIC GNRH NEUROANATOMY: PRIMATES

While the bulk of the neuroanatomical literature is available from rodents and ruminants, immunohistochemical and in situ hybridization experiments on monkeys and humans have revealed some interesting species‐specific anatomical characteristics of the GnRH neuronal system in primates. Results of these studies may become particularly useful for the understanding of the central regulation of human fertility and its disorders. This chapter aims to highlight some unique features of primate GnRH cells.

### 4.1 Presence of two GNRH genes in the primate genome

Reproduction in nearly all mammals is controlled by the mammalian form of GnRH decapeptide (mGnRH) encoded by the sole *GNRH1* gene. Unlike laboratory rodents, primates contain a second *GNRH* gene (*GNRH2)* in their genome.^109^ This gene is inactivated in the orangutan and the chimpanzee, whereas it remains fully functional in rhesus monkeys, humans and several other primates.^109^ In situ hybridization studies of the macaque brain localized *GNRH2* mRNA expressing neurons in the midbrain, hippocampus and the hypothalamic supraoptic, paraventricular and suprachiasmatic nuclei and the ARC.^110^ The oestrogenic induction of *GNRH2* expression in this species raised the intriguing possibility that, in some primates, GnRH‐II neurons may contribute to oestradiol‐positive feedback and the midcycle preovulatory LH surge.^110^ We note that the biosynthesis of the functional GnRH‐II peptide in mesencephalic and hypothalamic neurons of the rhesus monkey brain still awaits confirmation.^110^ Mapping the distribution of *GNRH2* expressing neurons in the human brain remains an interesting challenge for future research. The following sections will focus on the neuroanatomy of the primate GnRH‐I system.

### 4.2 GnRH neuron development in primates

As reported for different mammals, GnRH neurons in primates develop in the olfactory placodes and migrate to the brain prenatally.^18^ The detailed spatiotemporal profile of this migration in the human embryo has been characterized recently by the Giacobini laboratory.^111^ These authors confirmed the existence of a ventral migratory pathway also known in other species which carries ~2,000 neurons to the developing human hypothalamus to regulate reproduction after puberty. In addition, they discovered and reported a second dorsal route previously known only in monkeys^112^ whereby ~8,000 neurons migrated toward extrahypothalamic structures of the human brain.^111^ It is currently unclear where these neurons finally settle and whether they survive into adulthood. Interestingly, it has only been established recently that the number of extrahypothalamic GnRH neurons in the adult human brain is much higher than 8,000 (104,000–229,000 in different brain samples),^113^ suggesting that the majority of extrahypothalamic GnRH neurons in humans might be brain‐born, instead of originating from the olfactory placodes.^113^

### 4.3 Topography and morphology of GnRH neurons in the primate hypothalamus

**FIGURE 3:**
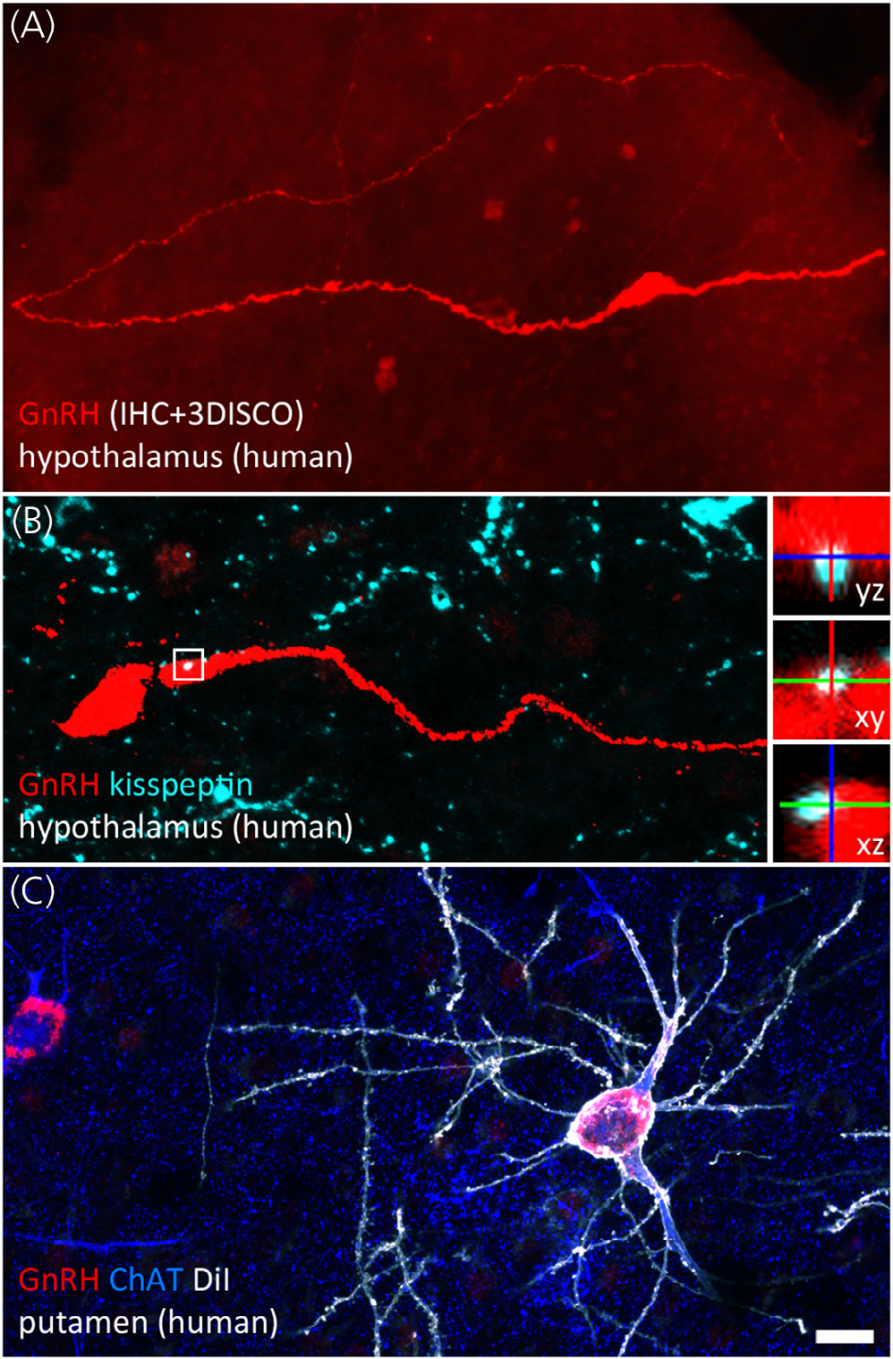
Hypothalamic and extrahypothalamic GnRH neurons of the adult human brain. (A) Hypothalamic GnRH neurons regulating reproduction are typically fusiform. In 1 mm‐thick slices made transparent with the 3DISCO clearing technology, lengthy GnRH dendrites can be followed occasionally for several millimetres. Dendrites may represent the main cellular compartment receiving afferent inputs. (B) Kisspeptin‐immunoreactive inputs to GnRH neurons (turquoise) from the infundibular (arcuate) nucleus convey information about circulating sex steroid levels. High‐power insets show orthogonal views of a neuronal apposition. (C) Unlike rodents, humans contain hundreds of thousands of GnRH‐immunoreactive (red) neurons in extrahypothalamic brain regions. The dendritic tree of GnRH cells in the putamen can be visualized post mortem using the lipophilic dye DiI (shown in white) delivered to the sections with the aid of a Gene Gun. Labelled GnRH neurons exhibit smooth surfaced dendrites and correspond to a subpopulation of cholinergic interneurons immunoreactive to choline acetyltransferase (ChAT; blue). Scale bar: 20 μm in (A) and (C), 16 μm in (B) (insets: 5 μm). Photograph courtesy of Dr Katalin Skrapits, Institute of Experimental Medicine, Budapest

The majority of the anatomical studies in the primate brain used immunohistochemistry to localize GnRH neurons.^113^, ^114^, ^115^, ^116^, ^117^, ^118^, ^119^ Perikaryon distribution in monkeys and humans was found to be even looser than in rodents. The large hypothalamic volume contains cell bodies from the septal‐preoptic to the retromammillary region. GnRH neurons were reported within the preopticoseptal region, diagonal band of Broca, lamina terminalis, periventricular and infundibular nuclei and the mammillary region.^115^, ^116^, ^119^, ^120^ Some investigators observed a few cell bodies in the ventromedial nucleus.^118^ Unlike in laboratory rodents, the OVLT contained relatively few GnRH cells.^119^ Morphologically, the majority of GnRH neurons in primates are fusiform and bipolar, with two processes emanating from the opposite poles of a thin soma^116^, ^119^, ^121^ (Figure 3A). A smaller subpopulation are multipolar, with triangular or rounded cell body.^118^, ^119^, ^120^ GnRH neurons were found to be considerably larger in adult human males (37× 14 μm) and in rhesus monkeys (28× 12 μm) than in bats (16 × 9 μm) or rats (16 × 6 μm).^50^ Information about the fine structure of primate GnRH neurons is limited to the subcellular compartments visualized with immunohistochemistry, in the absence of efficient cell filling and 3D neuronal reconstruction techniques to study dendritic arborization, filopodia, spines and network connectivity.

Rance and coworkers analysed the distribution of human GnRH neurons with in situ hybridization. Based on perikaryon size and labelling density, they classified *GNRH1* mRNA expressing neurons into three main categories.^122^ The highest levels of *GNRH1* mRNA signal were observed in small oval or fusiform (“type 1”) GnRH neurons in the medial basal hypothalamus, ventral POA and periventricular zone. These cells probably correspond to the bulk of hypothalamic GnRH neurons detectable with immunohistochemistry.^119^ Interestingly, in situ hybridization also revealed thousands of additional “type 2” and “type 3” *GNRH1* mRNA expressing neurons at various extrahypothalamic sites of the human brain.^122^

### 4.4 GnRH fibre projections

As described in different species,^50^, ^123^ several circumventricular organs, including the ME, the OVLT and the subfornical area, receive input from GnRH neurons in primates.^50^, ^116^, ^120^ As shown in monkeys, POA as well as MBH GnRH neurons contribute to the neuroendocrine regulation of the pituitary because both populations can be labelled by microinjection of retrograde tracers into the median eminence.^117^ The anatomical route of neurosecretory GnRH fibres in primates shows some important species‐specific features. Accordingly, the median eminence containing the neurohemal junction is positioned rostral to the infundibular stalk in laboratory rodents, whereas it is postinfundibular in primates.^124^ In humans, some neuroendocrine GnRH cell bodies can be more than a centimetre away from this site. Further, neuroendocrine GnRH axons all terminate within the external zone of the median eminence in rodents, whereas a considerable subset enter the infundibular stalk and some travel all the way down to the neurohypophysis in primates.^50^, ^121^, ^125^ In humans, these descending GnRH fibres are accompanied and occasionally contacted by other peptidergic fibres containing KP, NKB and Substance P (SP).^126^ GnRH secreted from these processes may have access to adenohypophysial gonadotrophs via the short portal veins.^125^ Neuroendocrine GnRH projections outside the blood–brain barrier are exposed to blood‐born substances which also explains the mechanism whereby systemic KP injection causes prompt LH secretory responses also in humans.^127^

Presence of nonhypophysiotropic GnRH fibres in the habenula, amygdala, hippocampus and around the mamillary bodies^50^, ^115^, ^119^, ^120^, ^128^ indicates that GnRH also serves as a neurotransmitter at hypothalamic and extrahypothalamic sites. This non‐neuroendocrine role is supported by the electron microscopic demonstration of symmetrical synapses between GnRH‐immunoreactive axons and GnRH‐immunoreactive perikarya and dendrites in monkeys^129^ and by light microscopic evidence for direct contacts between GnRH containing axons and SP neurons in humans.^130^

### 4.5 Afferent regulation

Various neurotransmitter systems contributing to the neuronal regulation of human GnRH neurons have been reviewed recently.^131^ Neuropeptides detected previously with light‐ or confocal microscopy in afferent contacts to GnRH perikarya and dendrites include neuropeptide Y, SP, galanin, corticotropin releasing hormone, KP (Figure 3B), NKB, endorphins, enkephalins, Dyn, RF‐amide related peptides, GnRH, orexins and melanin concentrating hormone.^131^, ^132^, ^133^ The incidence of such contacts is region‐dependent and, as a general tendency, higher to GnRH neurons in the infundibular versus the POA.^132^ Similarly, GnRH neurons in the ARC of monkeys receive higher numbers of synapses than those in the POA.^134^ The number of appositions is also sex‐ and age dependent. GnRH neurons receive more KP‐immunoreactive and NKB‐immunoreactive appositions in aged versus young men^135^ and in postmenopausal women versus age‐matched middle aged/aged men.^136^ It would be difficult to determine if these differences reflect the anatomical plasticity of afferent connections or simply a better detectability of higher antigen levels in aged individuals due to lower sex steroid and negative feedback levels.

The cell bodies and proximal dendrites of GnRH neurons in different species receive relatively few classical synapses, as also demonstrated with electron microscopy in monkeys.^134^ The number of synapses decreases following ovariectomy and can be restored by steroid replacement.^134^ These observations somewhat contrast with the opposite changes in the number of KP and NKB contacts detected on human GnRH neurons with light microscopy^136^ and also imply that synaptic specializations may often be absent from peptidergic afferent contacts.

In addition to various neuropeptides, fibres innervating human GnRH neurons contain the classic neurotransmitters histamine, catecholamines, γ‐aminobutyric acid and glutamate.^131^, ^132^, ^137^, ^138^

A substantial proportion of neuronal inputs may target the dendritic compartment of primate GnRH neurons, as it was proposed in mice. While the putative existence of “dendrons” in primates needs to be established, GnRH neurons possess lengthy dendrites which are likely to receive and integrate a substantial number of synaptic signals. The primary importance of dendritic inputs is supported by the observation that 75%–80% of the orexin‐ and melanin concentrating hormone‐containing afferents target the dendritic rather than the somatic compartment of human GnRH cells.^133^ Similarly, each 100 μm dendritic segment receives similar numbers of light microscopic GABAergic and glutamatergic appositions as does the human GnRH perikaryon.^139^

### 4.6 Paracrine regulation of GnRH fibres

Many thin GnRH‐immunoreactive processes in the primate hypothalamus show the typical appearance of varicose axons and this fibre phenotype becomes dominant around the human portal capillaries of the median eminence and in the neurohypophysis.^126^ Nonsynaptic communication between KP and GnRH fibres within the median eminence was proposed to represent the primary mechanism whereby endogenous KP regulates GnRH neurons in monkeys^140^ and similar axoaxonal contacts were detected later in the human median eminence and hypophysial stalk.^126^

### 4.7 GnRH neuron cotransmitters in primates

Although little is currently known about the classical and peptide neurotransmitters of primate GnRH neurons, existing species differences deserve some discussion. Galanin, which is cosynthesized in a sexually dimorphic and oestrogen‐dependent manner in GnRH neurons of rats,^141^, ^142^, ^143^ seems to be absent from human GnRH neurons.^144^ Similarly, while GnRH neurons of rats exhibit immunoreactivity to γ‐aminobutyric acid prenatally^145^ and the glutamatergic marker type‐2 vesicular glutamate transporter (VGLUT2) in adulthood,^146^ earlier studies using confocal microscopy failed to reveal amino acid neurotransmitters or the more readily detectable vesicular amino acid transporter markers^139^ in human GnRH neurons.

Somewhat unexpectedly, a large subset (~35%) of hypothalamic GnRH neurons in the adult human brain was found to be immunoreactive to the cholinergic phenotype marker choline acetyltransferase. The cholinergic phenotype, which has not been reported in other species yet, develops prenatally after human GnRH neurons enter the brain and is typical both in hypothalamic and extrahypothalamic GnRH neurons.^113^

### 4.8 Ovarian oestrogen signalling to primate GnRH neurons

Oestrogen feedback in primates appears to involve a stronger pituitary component than in laboratory rodents. For example, a study using graded oestrogen infusion to postmenopausal volunteers, followed by 18FDG positron emission tomography, came to the conclusion that positive estrogen feedback takes place dominantly in the pituitary, whereas negative feedback on LH is primary hypothalamic.^147^ Another study using diffusion MRI and MR spectroscopy techniques identified transient microstructural and metabolic changes in the human female hypothalamus (but not in the thalamus) following the withdrawal of a combined low‐dose oestrogen‐progesterone treatment. This treatment paradigm is thought to serve as a model for hypothalamic plasticity during negative feedback in the early follicular phase of the menstrual cycle.^148^ Reduced oestrogen levels in postmenopausal women cause robust neuronal hypertrophy of estrogen‐sensitive neurons in the human infundibular region; hypertrophied neurons are devoid of GnRH mRNA.^149^ Although a subpopulation of human GnRH neurons are immunoreactive to the beta oestrogen receptor isoform,^150^ oestrogen feedback to GnRH neurons is likely to be primarily indirect, in accordance with the conclusion drawn from results in other species.

The hypothalamic ARC (also called infundibular nucleus in the human) has long been known as the primary site of sex steroid feedback in primates. The loss of estrogens in postmenopausal women causes the hypertrophy of neurons expressing ER‐α,^149^ SP,^151^ NKB,^151^ KP^152^ and proDyn^153^ in this area. The homologous “KNDy” neurons (expressing KP, NKB and Dyn) are thought to be important players of negative oestrogen feedback and GnRH/LH pulsatility in other species, but some primate‐specific characteristics deserve to be mentioned. First, human KP neurons exhibit a unique pattern of neuropeptide colocalization. Differences from their rodent KNDy neuron counterparts include presence of SP and cocaine‐ and amphetamine regulated transcript and absence of Dyn and galanin in human KP neurons.^154^ Second, the human KP system exhibits an unusually robust sexual dimorphism and aging‐dependent plasticity, as reviewed recently.^155^ Third, while the somatodendritic compartment of GnRH neurons in mice receives only sparse innervation from KNDy neurons of the MBH,^156^ at least ~10%–30% of the KP input to human GnRH neurons originates from this site.^135^, ^157^

Positive oestrogen feedback to GnRH neurons in rodents if thought to be accounted by KP neurons located in the rostral periventricular area of the third ventricle mediate These neurons contain estrogen receptors, innervate GnRH neurons, express FOS during the LH surge and are substantially more numerous in females than in males.^158^ Surgical isolation of the MBH in rhesus monkeys did not prevent spontaneous ovulation and the oestrogen‐induced LH surge, giving support to the prevailing view that this region accounts for positive oestrogen feedback in primates.^159^, ^160^ The POA/rostral hypothalamus of humans and monkeys contains KP cell groups which anatomically resemble the homologous rodent neurons.^155^, ^161^, ^162^ These cell groups may also contribute to positive feedback because they exhibit increased FOS immunoreactivity^163^ and *KISS1* mRNA expression^164^ in response to a surge‐inducing oestradiol regimen in monkeys. Further, *KISS1* also seems to be regulated positively by oestrogens in the human rostral hypothalamus, which was concluded from the reduced number of KP‐immunoreactive neurons after menopause.^161^

### 4.9 Functional networking of GnRH neurons

The rhythmic LH secretory bursts from the anterior pituitary requires a coordinated secretory activity of GnRH neurons. Although the mechanisms underlying this coordination are still not entirely understood, regulation of neurosecretory GnRH preterminals/terminals by volume transmission may play an essential role in this process also in primates.

Classical synapses interconnecting GnRH neurons in nonhuman primates^129^ may also contribute to the synchronized secretion of a larger GnRH neuron population. Frequent axosomatic and axodendritic contacts were also reported between human GnRH neurons.^119^ Witkin et al. described an unusual syncytium‐like connection of neighbouring GnRH neurons in which GnRH‐GnRH contact sites appeared to exhibit cytoplasmic confluence at the ultrastructural level.^32^ Equivalent to this could be the end‐to‐end dendritic continuity observed between close pairs of GnRH neurons at the light microscopic level.^119^ Somewhat against the idea of cytoplasmic continuity is the lack of evidence for dye coupling between murine GnRH neurons during their studies with slice electrophysiology. We note that the resolution of light‐ or confocal microscopy in most anatomical studies on the primate GnRH system was unable to visualize intertwined dendrites and dendritic bundles similar to those described in mice.^165^

### 4.10 The extrahypothalamic GnRH neuron population of primates

While GnRH neurons in adult laboratory rodents are located nearly exclusively in septal, anterior hypothalamic and preoptic areas and subserve functions related to reproduction, a handful of anatomical studies on primates identified large numbers of additional *GNRH1* mRNA expressing and/or GnRH immunoreactive neurons at extrahypothalamic sites not related closely to reproductive regulation. These sites included several basal ganglia, the basal forebrain and the amygdala.^112^, ^118^, ^122^, ^166^, ^167^ Initial interest toward these cells somewhat decreased because extrahypothalamic GnRH neurons in the developing monkey brain could not be immunostained with all GnRH antibodies and they also showed immunoreactivity to metalloendopeptidase which can cleave GnRH at the Tyr5‐Gly6 position.^166^ These observations raised the possibility that extrahypothalamic GnRH neurons of primates contain the GnRH degradation product GnRH1‐5, instead of the bona fide GnRH decapeptide.

Recently the Hrabovszky laboratory has provided a more comprehensive characterization of extrahypothalamic GnRH neurons located in the human basal ganglia and basal forebrain^113^ which correspond to the large and round “type 3” neurons expressing intermediate levels of *GNRH1* mRNA between the heavily labelled “type 1” neurons of the hypothalamus and the lightly labelled small, oval “type 2” neurons reported previously in the septum, dorsal POA, bed nucleus of the stria terminalis and amygdala.^122^ Immunohistochemical studies combined with stereology revealed that these regions contain 150,000–200,000 GnRH‐immunoreactive neurons. 82% of labelled cells were observed in the putamen, 5.5% in the accumbens nucleus, 4.9% in the caudate nucleus, 3.5% in the basal nucleus of Meynert, 1.8% in the globus pallidus, 1.3% in the ventral pallidum and 0.8% in the bed nucleus of the stria terminalis.^113^ The neurons were detectable with a series of different GnRH and GnRH‐associated peptide antibodies and high performance liquid chromatography/tandem mass spectrometry (HPLC‐MS/MS) analysis of putamen extracts revealed presence of the GnRH decapeptide and its dominance over its degradation product GnRH1‐5. These studies established that extrahypothalamic GnRH neurons express the cholinergic marker choline acetyltransferase and correspond to subsets of previously known cholinergic interneuron populations in the putamen and cholinergic projection neurons in the basal nucleus of Meynert^113^ (Figure 3C).

The functional significance of GnRH cosynthesis in cholinergic systems is difficult to study in the absence of appropriate rodent models. Deep transcriptome profiling of cholinergic interneurons and spiny projection neurons, which represent main target cells of the former, established that the *GNRHR1* transcript is only expressed in cholinergic interneurons. Therefore, at least in the human putamen, GnRH appears to act on GnRHR1 autoreceptors to regulate higher order nonreproductive functions.^113^

## 5 UNRESOLVED QUESTIONS AND FUTURE RESEARCH OPPORTUNITIES

Comparative and complementary neuroanatomical approaches across mammalian species have led to a greater understanding of how GnRH neurons orchestrate reproductive function. While rodent models provide a wealth of readily available transgenic neuroscience tools that can be harnessed, sheep models have the advantage of being able to couple neuroanatomical findings with GnRH secretion dynamics and seasonal reproductive function, and primates may be our best models of the human condition. Ongoing neuroanatomical research across all of these species is required if we are to fully understand GnRH neuron biology.

Over the past two decades, we have seen technical advances in transgenics, viral mediated transduction tools, tissue clearing and expanding methods and image analysis tools that have revolutionised our ability to assess information about GnRH neuron anatomy and network connectivity. Transgenic expression of GFP,^34^, ^76^, ^77^, ^78^, ^168^ or Cre recombinase^38^, ^67^, ^169^ in rodent GnRH neurons has been instrumental in visualising regions of the GnRH neuron that are difficult to detect with antibodies detecting GnRH peptide alone and for driving the expression of circuit tracing and functional measurement tools in a cell‐specific way. In addition to enabling visualisation, GnRH neurons expressing fluorescent markers have provided the opportunity to sort and isolate the hypothalamic GnRH neuron populations in the postnatal brains of both mice^170^ and rats.^96^ The expression of calcium indicators has enabled researchers to visualise the activity of GnRH neuron elements in real time and in situ.^67^, ^171^, ^172^, ^173^, ^174^ Tissue clearing and advanced microscopy methods in rodents, sheep and primates have made it possible to visualise whole GnRH neurons in detail and whole GnRH neuron populations in situ throughout the brain.^60^, ^111^, ^175^ While we have learned a great deal through these advances over the past several decades, many important questions remain to be answered that will probably require additional technological advances. Below are just some of the remaining questions that will be interesting to target going forward.

1. Is the dendron described in rodents a universal feature of mammalian GnRH neurons? The ability to visualise and functionally assess the rodent dendron is currently dependent upon transgenic approaches that enable visualising or targeting individual GnRH neurons. Will transgenic approaches or other methodologies become available that will allow us to visualise the full extent of individuals GnRH neurons in other species?
2. What is the role of extrahypothalamic GnRH signalling? While it is known that GnRH neurons across species possess subpopulations of nonhypophysiotropic cells and that GnRH neurons project to several brain regions outside of the median eminence, the role of central GnRH signalling is largely unknown. Can functional neuroscience tools such as opto‐ or chemogenetics help us to identify the roles of these extrahypothalamic pathways?
3. Do GNRH2 neurons play an essential role in the regulation of fertility in primates? To date, hypothalamic GNRH2 neurons were only studied with in situ hybridization in Rhesus monkeys. The estrogenic regulation of their GNRH2 mRNA expression raises the intriguing possibility that these cells contribute to the regulation of mammalian fertility in humans and some other primates.
4. How does transcriptome plasticity of GnRH neurons regulate reproductive physiology and pathology? Newly available single‐cell RNA‐seq technologies open the way for deep transcriptome profiling of GnRH neurons from laboratory animals and even from post mortem human brains.
5. Can in vivo monitoring of cellular activity be engineered across species? The advances in nanotechnology and genetic modification enable following neurons, dendrites, and spines in living organisms. Extension of these approaches to GnRH systems in multiple species could fill the gaps in our knowledge of species‐specific differences in GnRH regulation.

## AUTHOR CONTRIBUTIONS

**Rebecca E Campbell:** Conceptualization; funding acquisition; writing – original draft; writing – review and editing. **Lique M. Coolen:** Conceptualization; funding acquisition; writing – original draft; writing – review and editing. **Gloria E. Hoffman:** Conceptualization; funding acquisition; writing – original draft; writing – review and editing. **Erik Hrabovszky:** Conceptualization; funding acquisition; writing – original draft; writing – review and editing.

### PEER REVIEW

The peer review history for this article is available at https://publons.com/publon/10.1111/jne.13115.

## Data Availability

Data sharing not applicable to this article as no datasets were generated or analysed during the current study.
